# Further understanding on osteopetrotic femoral fractures: a case report and literature review

**DOI:** 10.1186/s12893-021-01107-4

**Published:** 2021-03-06

**Authors:** Haiqi Ding, Hongjiang Chen, Haiming Lin, Jiankun Xu, Zhonglian Huang, Wensheng Li, Jun Hu

**Affiliations:** Department of Orthopaedics, First Affiliated Hospital of Shantou University Medical College, Shantou, 515041 People’s Republic of China

**Keywords:** Osteopetrosis, Intermediate autosomal recessive osteopetrosis, Peritrochanteric fractures, Subtrochanteric femur fractures, Dynamic hip screw

## Abstract

**Background:**

Osteopetrosis is a genetic disease characterized by defects in osteoclast formation and function. There were a few cases of subtrochanteric femur fractures treated with dynamic hip screw (DHS) in patients with osteopetrosis, but unfortunately the healing outcome was rather poor.

**Case presentation:**

We present our experience for treating a patient with intermediate autosomal recessive osteopetrosis (IRO) suffering from subtrochanteric femur fracture. In this case, we successfully used dynamic hip screw (DHS) internal fixation through meticulous preoperative planning and postoperative care, as well as application of surgical techniques. The patient displayed stable internal fixation with no limitation of activities during follow-up for 15 months. In addition to this case, a review of previous case reports showed an increasing number of case reports demonstrating that surgical treatment-related complications could be avoided preoperatively, intraoperatively, and postoperatively.

**Conclusion:**

DHS for this patient, who suffered from subtrochanteric fractures with osteopetrosis, was successfully implemented. In the light of a comprehensive literature review, preoperative planning, surgical techniques, and postoperative rehabilitation care can significantly reduce the complications.

## Background

Osteopetrosis is a genetic bone disease characterized by defects in osteoclast function or a reduction in osteoclast number that results in defective bone resorption [1, 2], and is clinically characterized by increased bone mineral density and bone deformities [3, 4]. As a familial trait, osteopetrosis can be divided into autosomal recessive osteopetrosis (ARO), intermediate autosomal recessive osteopetrosis (IRO), autosomal dominant osteopetrosis (ADO) and X-linked osteopetrosis (XLO) [4, 5]. According to severity, it can be divided into a "malignant" autosomal recessive infant type, "benign" adult autosomal dominant type, and intermediate type. The malignant type is common in infancy and deteriorates rapidly, leading to death in the first few years [6]. Patients with the benign type or intermediate type have a normal life span, but have a higher incidence of fractures and long bone deformities [6, 7]. With significant mortality and teratogenicity, osteopetrosis imposes a considerable psychological and economic burden on families. Fortunately, in the wake of development of economies and improvements in medical technology, many early diagnosis techniques and various treatment strategies have been reported, and surgical treatments are also increasingly adopted for treating such challenging conditions.

Most patients with osteopetrosis are hospitalized with fractures, and in the past, it was thought that the majority of these fractures could successfully heal via conservative treatment, whereas surgical fixation frequently resulted in clinical failure [8]. Additionally, operative treatment of osteopetrotic fractures has been generally reported as intractable and accompanied by many complications [9, 10]. However, in last decade, with the development of surgical instruments and improvement of surgical techniques, various surgical treatment modalities and techniques have been reported for osteopetrotic fractures [11–15].

We report a case using dynamic hip screw (DHS) fixation to treat an adult patient with IRO and a subtrochanteric fracture. Furthermore, we reviewed previous case reports and found that preoperative assessment, flexible use of surgical techniques and postoperative professional rehabilitation training and nursing can greatly reduce the risk of surgical complications.

## Case presentation

**Fig. 1:**
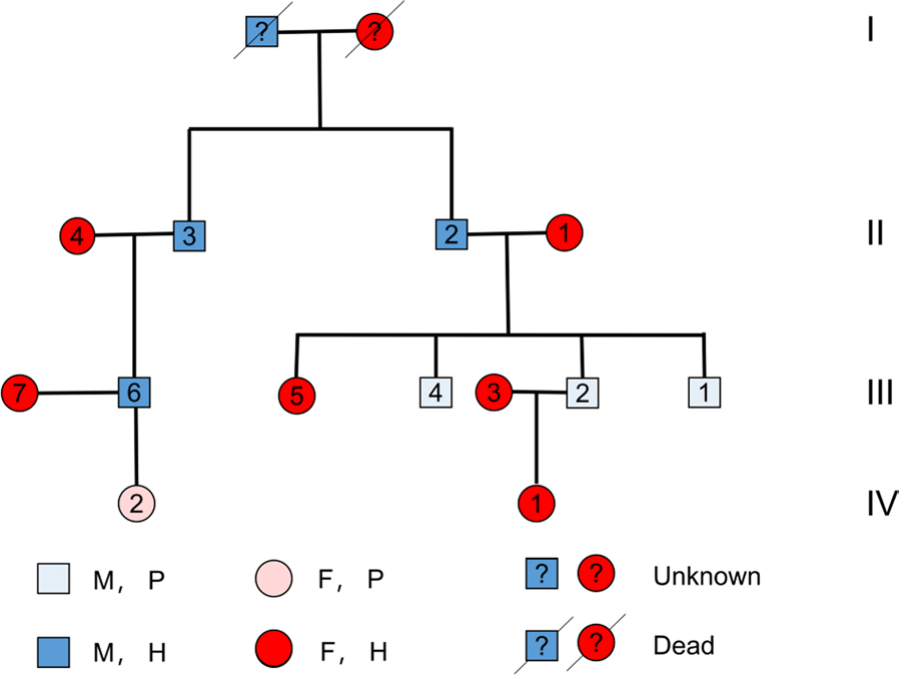
Pedigree chart of the patient’s family. III-2 is the patient. *F* female, *M* male, *P* patient, *H* health

**Fig. 2:**
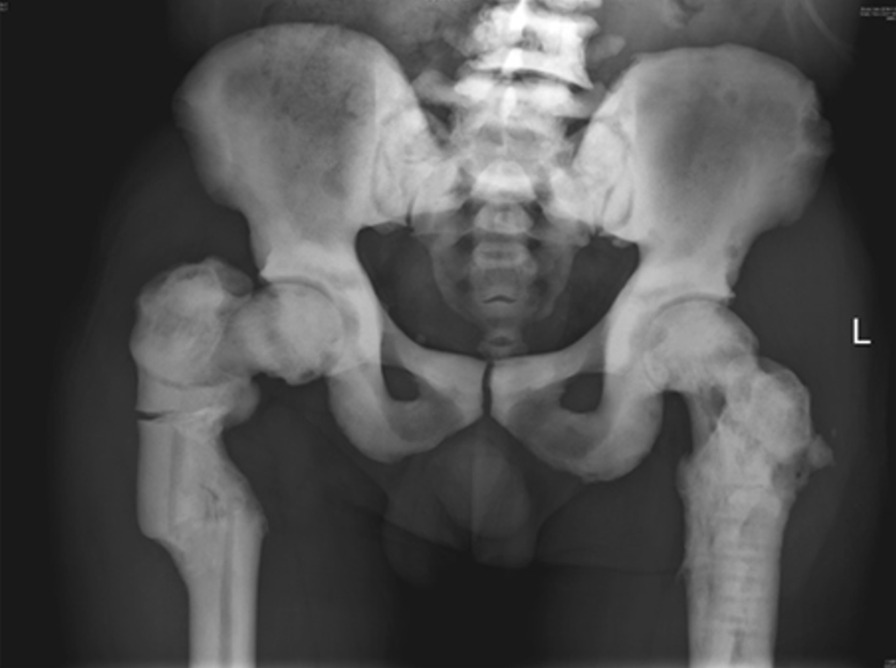
Radiograph taken three days before the first operation

A 52-year-old man, with a known history of osteopetrosis and previous fractures in his bilateral femurs and right tibia, was referred for treatment of a right subtrochanteric femur fracture in January 2018. His medical history is summarized in Table 1, which shows that the patient suffered from chronic osteomyelitis of tibia after traction treatment of tibial tubercle. A pedigree chart was drawn according to his family history (Fig. 1). In the chart, III-2 is the patient, and his previous generation, sister, cousin and his wife had no similar medical history, whereas two of his brothers suffered from osteopetrosis, and one of them presented with osteomyelitis of the mandible. Additionally, his daughter was healthy, but his niece suffered from osteopetrosis. When admitted to the hospital, he presented with severe functional limitation of the right hip and pain, and his radiograph (Fig. 2) showed that the lateral transverse fracture of the right femur was mildly displaced with a dense sclerotic line. In addition, the bone cortical density of the pelvis and the bilateral femur increased, and the proximal regions of bilateral femurs were deformed, with rough and irregular bone cortex, as well as narrowed medullary cavity, and signs of "sandwich vertebrae" were seen on the lower lumbar vertebrae. Additionally, laboratory testing showed mild anemia and slight hypocalcemia. According to family history, we judged the genetic pattern of the patient to reflect autosomal recessive inheritance. Together, his condition was diagnosed as IRO, accompanied by chronic osteomyelitis, mild anemia and hypocalcemia.

**Table 1:** Brief introduction of fractures (*R* right; *L* left, *F* femur, *T* tibia)

Age	Fracture location	Treatment	Follow-up
43	R, F	Bone traction	Malunion, chronic osteomyelitis of right tibia
46	L, F	Plate, screw and cortical strut	Union at 2 years
49	R, T	Plate, screw and cortical strut	Union at 2 years
52	R, F	DHS	Hardware failure at 3 months post surgery
53	R, F	DHS	Obtained clinical healing at 12 months

*DHS* dynamic hip screw

**Fig. 3:**
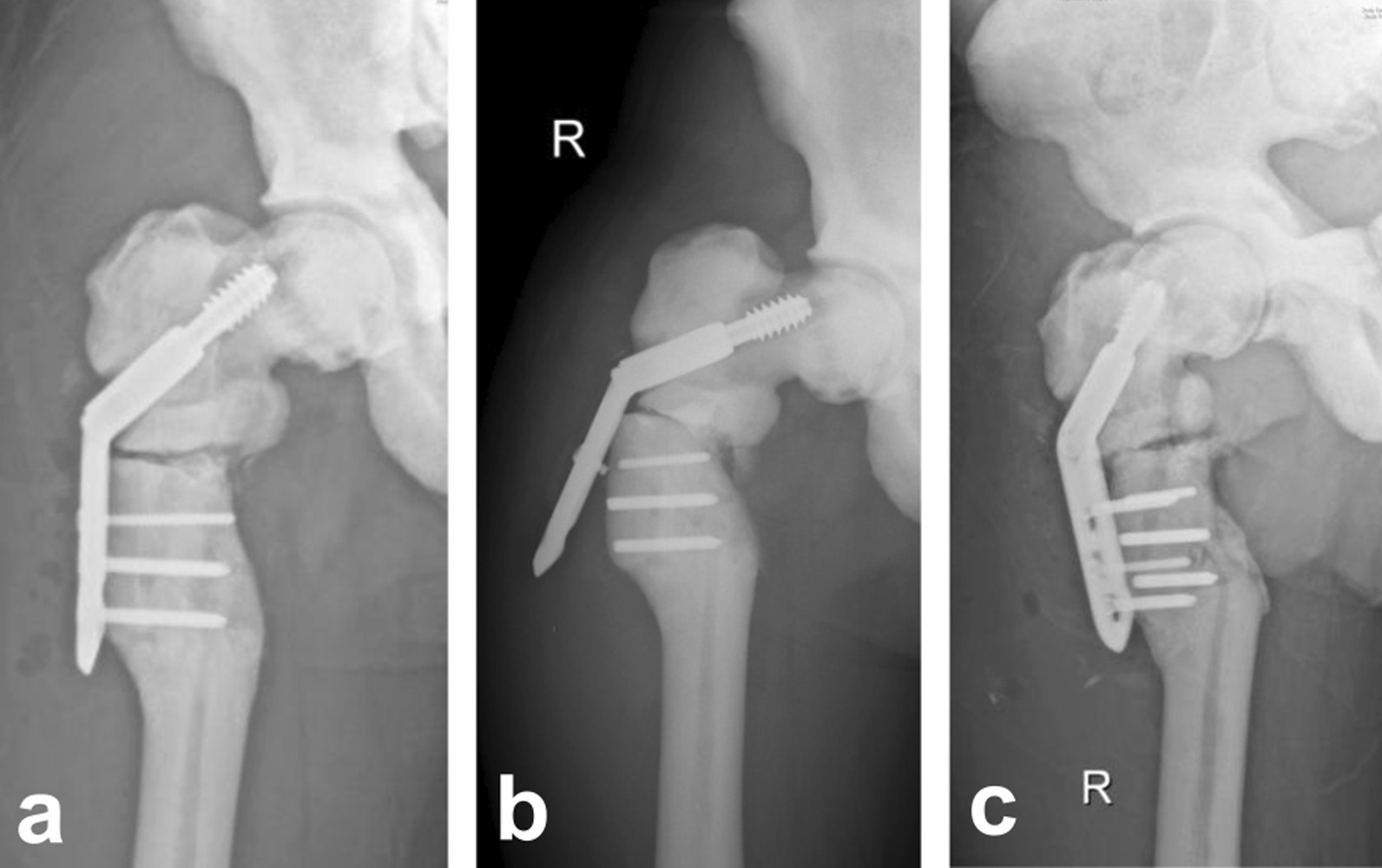
X-rays. **a** 1 week after the first operation. **b** Screw fracture 3 months after the first operation. **c** 2 days after the repeated DHS fixation

Before the operation, we evaluated the patient's bone condition and decided to treat the fracture using internal fixation with DHS. Importantly, we made sufficient preparation for the operation to deal with potential adverse events, such as drill bit fracture and bone necrosis. During the operation, we exposed the fracture site and found that the end presented a solid white amorphous appearance. As speculated, no medullary cavity existed in the femur. Two drill bits were used to create holes before inserting the screws with extreme care so as not to shatter the femoral shaft, and we chose a short hip screw with a length of 65 mm. The femur was resistant to drilling and it was done slowly under constant irrigation with ice-cold saline and with repeated cleaning of the drill bit. Eventually, we took triple longer duration than usual to complete this operation. X-ray images of our patient's pelvis, right hip, and right femur were taken 1 week after the operation (Fig. 3a) and showed satisfactory alignment of the fracture had occurred. Unfortunately, the right femoral internal fixation screws fractured (Fig. 3b), caused by a careless sprain 3 months after the operation. After re-admission, we performed a repeat of the DHS internal fixation for postoperative screw fracture in a right subtrochanteric fracture, and postoperative X-ray examination showed that the fracture was aligned (Fig. 3c).

**Fig. 4:**
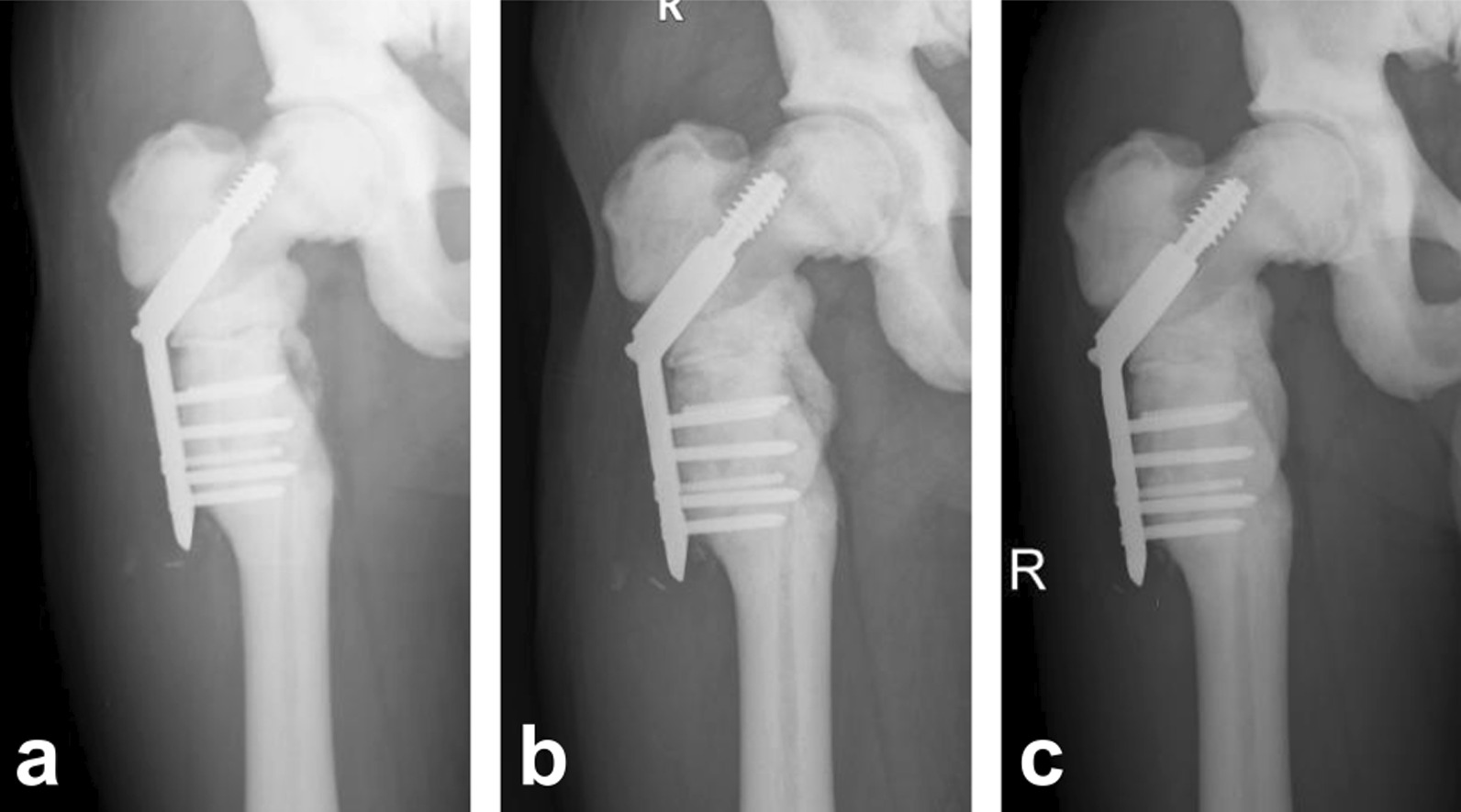
X-rays. **a** 3 months after revision. **b** 6 months after the revision. **c** 12 months after revision

Drawing a lesson from the previous failure of internal fixation, a double hip herringbone brace was fixed for 3 months to prevent the internal fixation from breaking again. Our patient returned for follow-up 3 months after the revision, at which time the radiograph still failed to demonstrate any healing progression (Fig. 4a). A blurred fracture line and callus formation were observed at 6 months after the revision (Fig. 4b). However, good evidence of callus formation and fracture healing was demonstrated by the radiograph at 12 months after the revision surgery (Fig. 4c). At 15-months follow-up, there were no clinical signs of infection, and laboratory tests remained within the normal range, except for a minor abatement in hemoglobin and serum calcium. At present, the patient displays stable internal fixation, with no limitation of activities, and is pain-free.

## Discussion and conclusion

Osteopetrosis is a disease that is characterized by metabolic disorder with diminished bone resorption. With an abnormal calcium metabolism, a decrease of the hematopoietic tissue and impaired leukocyte function, patients with IRO present with manifestations such as fracture, osteomyelitis, mild anemia and hypocalcemia [16, 17]. Due to the brittle nature, most patients with IRO generally are admitted to the hospital with a fracture, and the most common fracture location is the femur [8]. Furthermore, albeit difficult and related to many complications, surgical treatment can provide strong fixation of the fracture so that the patient can initiate functional rehabilitation training earlier, which is of great benefit for fracture healing and functional restoration. In fact, no matter whether conservative treatment or surgical treatment is used, continued risks of nonunion, varus malunion and re-fracture exist and are determined by the pathogenesis of osteopetrosis. Even in other fractures, complications such as hardware failure and infection may still occur. Additionally, the indications for conservative treatment are limited, and there also exist complications with conservative treatment, including nonunion, coxa vara deformity, pressure ulcer, hypostatic pneumonia and deep venous thrombosis. Among them, coxa vara deformity can lead to dysfunction, and ultimately requires a valgus osteotomy. Therefore, the urgent problem to be solved in the clinic is how to reduce the risk of complications of surgical treatment and achieve better therapeutic results.

With regard to the operative treatment of femoral fractures in patients with osteopetrosis, several related case reports and small-scale case series are available in the literature (Table 2) in which 41 cases of surgical treatment of osteopetrotic fracture have been reported, including 6 cases of femoral neck fractures, 3 cases of femoral shaft fractures and 32 cases of peritrochanteric fractures. In a total of 50 operations, there was a 6.00% nonunion rate and a 6.00% infection rate, and all infected fractures failed to achieve union (Table 2). In this cohort, the rate of hardware failure was 16.00%, and the incidence of periprosthetic fracture was 6.00%. After further analysis, we found that with the progress of surgical techniques and the accumulation of clinical experience in the treatment of osteopetrosis-associated fracture, reports of cases with complications of surgical treatment decreased significantly. The cases published before 2005 consistently reported complications associated with operative treatment, with a 54.55% complication rate, a 27.27% reoperation rate and an 18.18% nonunion rate. In contrast, in the cases reported after 2005, the rate of complications decreased to 21.05%, the rate of reoperation decreased to 10.53% and the incidence of nonunion was 0. As the difficulties encountered in reported cases have promoted the development of safe and efficacious techniques, the risks of surgical complications, such as fracture, osteonecrosis, implant loosening and infection, have been and will continue to be greatly reduced.

**Table 2:** Published studies on operative treatments of osteopetrotic femoral fractures

Study	Operation time	Age (years)	Gender	Fracture location	Treatment	Complications	Status at last follow-up
Kleinberg [6]	1954	35	M	L, p	Plate and screw	Hardware failure	Union
Yang et al. [20]	1980	21	F	L, p	Jewett nail	Hardware failure	Union at 12 months
		52	F	P	Holt nail plate	None	Union at 2 years
Ashby [21]	1992	49*	F	L, P	Zickel nail	Fragmentation of distal fragment with nail placement, placed cerclage wires	Union
		52*	F	L, P	THA after nail removal	None	Full weight bearing
		55*	F	R, P	THA	Periprosthetic fracture	Union at 10 months
		61**	F	L, P	Nail plate device	None	Union
		69**	F	R, P	Nail plate device	Infection	Nonunion
		70**	F	R, P	THA after 8 months	Nonunion	Nonambulatory
De Palma et al. [22]	1994	27	M	R, P	Jewett plate	Union, removed plate at 1 year, refractured, dynamic compression plate	Union
Armstrong et al. [23]	1999	N = 4	/	FN	Pins/compression screw	None in there and one treated with pins in 6 months	Union
		N = 3	/	P	Nail plate/compression screw plate	None	Union
		N = 2	/	P	ORIF	None in one and nonunion in another	One united at 6 months and nonunion in another
		31	M	P	Blade plate	None	Union at 16 weeks
Rolauffs et al. [24]	2002	39	M	R, FN	Parallel screws	Hardware failure, infection	Girdlestone
Su et al. [25]	2003	29	M	L, P	ORIF	None	Union
Zhang et al. [26]	2004	44	M	L, FN	THA	Periprosthetic fracture four weeks later	Union at 16 weeks
Chhabra et al. [9]	2005	22***	F	L, P	DHS revision after prior jewett nail	Infection	Infection, nonunion
		22***	F	R, P	DHS	Hardware failure	Pullout, nonunion
		41****	F	L, P	Kuntscher nail	Hardware failure	Union 2 months, rod removed at 6 months
		42****	F	L, P	Kuntscher nail	None	Union
		45****	F	R,P	Kuntscher nail	None	Union
		42	M	R, P	Proximally locked intramedullary nail	None	Union at 2 months
Bhargava et al.[11]	2009	48	F	B, S	Locking plate	Delayed union	Full weight bearing at 3 years
Cadosch et al. [27]	2009	37	M	R, P	Intramedullary nail	None	Union at 6 months
Kumar et al. [28]	2012	45	M	B, P	DHS	None	Union at 11 months
Golden and Rodriguez [29]	2010	27	M	B, P	Dynamic Condylar Screw	None	Full weight bearing at 3 years
Amit et al. [13]	2010	35	F	R, P	Locking plate	Incomplete stress fracture	Union at 23 weeks
		38	F	L, P	Locking plate	None	Union at 21 weeks
Somohata et al. [30]	2011	61	F	R, P	Hemiarthroplasty	None	Full weight bearing at 2 years
Kunnasegaran et al. [31]	2011	38	M	L, P	Plate-screw and total hip replacement	Hardware failure	Partial weight bearing at six weeks
Sen et al. [32]	2013	Mean 26	4 M/1 F	P	Locking plate	None	Union at 3 months
Huang et al. [33]	2013	23	F	B, S	Bilateral plate-screw	None	Unspecified
Kumbaraci et al. [34]	2013	21	F	B, P	Intramedullary nail(PFNA)	None	Union at 12 months
Matar et al. [35]	2014	12	F	L, S	Plate-screw	Unspecified	Unspecified
Huang et al. [14]	2014	67	M	L, P	Plate-screw (LISS)	None	Union at 12 months
Behera et al. [36]	2016	8	M	R, P	DHS	None	Union at 12 weeks
Seyfettinoglu et al. [15]	2016	49	F	L, P	Intramedullary nail (PFNA) and plate-screw	Hardware failure	Union at 12 months

*F* female, *M* male, *B* bilateral, *R* right, *P* peritrochanteric, *L* left, *S* femoral shaft, *FN* femoral neck, *THA* total hip arthroplasty, *ORIF* open reduction internal fixation, *DHS* dynamic hip screw, *LISS* less invasive stabilization system

^*^, **, ***, ****Represent a patient separately

We realize that traditional fracture treatment of osteopetrosis is associated with numerous complications that can be avoided by preoperative analysis and planning. According to Table 2, for osteopetrosis-associated femoral fractures, implants such as DHS, dynamic condylar screws (DCSs), intramedullary nails (IMNs), proximal femoral anti-rotation intramedullary nails (PFNAs), locking compression plates (LCPs) and total hip arthroplasty (THA) have been reported. Among them, inserting a plate and screw is the most commonly used surgical method for femoral shaft fracture, and the surgical methods for femoral neck fractures include pins, compression screws, parallel screws and THA, although almost all of the above operations have been reported for the treatment of peritrochanteric fractures. A case series reported by Chhabra et al. describes the long-term management of nine osteopetrotic femoral fractures in three patients, and demonstrated that the strategies of operative treatments depend entirely on the bone condition of the patient [3]. In our study, preoperative evaluation found that occlusion of the medullary cavity and femoral malformation limited the application of intramedullary fixation, so we finally decided on DHS fixation. At the same time, two sets of surgical instruments were prepared to be used alternately, along with sterile ice-cold saline irrigation for cooling down, to solve the problem of overheating the drill, which aided the successful completion of the operation. Together, preoperative evaluation, preparation and selection of surgical methods are crucial to reduce the incidence of fixation failure and important for completing the operation.

Osteopetrosis introduces technical limitations when drilling, reaming, or inserting implants, which can be minimized with special techniques. Due to resistance to drilling and reaming, the heat generated by long-term drilling friction will lead to osteonecrosis, and dulling, even fracture of the drill bits. Brittleness of the bone may lead to intraoperative fracture. In addition, the operation time must be extended, and the incidence of osteomyelitis and bone nonunion increases. According to suggestions from Bhargava et al. [11], Nampei et al. [12], Amit et al. [13], Ramiah et al. [18] and Matsuno et al. [19] and in combination with our surgical experience, techniques for performing the operation efficiently and safely can be summarized (Table 3).

**Table 3:** Intraoperative challenges and solutions

Challenges	Solutions
Drilling skills	Spaced cycles with low-speed drilling or use high-resistance and high-speed electric drill bits
Intra-operative fracture	Avoid inappropriate violence and use of hammers
Temperature control	Continuous cooling with saline, frequent change of drill bits
Infection	Strict aseptic operation, control of operation time, preventive use of drugs as necessary
Hip lag screw	Reducing length of drilling, tapping and inserting screws, regular cleaning of tap and screw tract
Lateral plate	Fully tapping all holes before screw insertion

Considering that these patients are at greatest risk for surgery-related complications or further injury, avoiding trauma and postoperative rehabilitation is particularly important throughout the postoperative course. In our study, the internal fixation screw fractured (Fig. 3) 3 months after the previous operation due to a rotational force, but ultimately, healing was observed and the patient formed a stable internal fixation with no limitations of activities after the repeated DHS fixation. Moreover, postoperative professional functional training can promote functional recovery and avoid deep venous thrombosis. In addition, incision care can reduce the risk of postoperative infection. At the same time, calcium supplementation and the use of drugs to promote osteogenesis can also help fracture healing.

Nevertheless, accumulating knowledge has enabled the development of safe and efficacious techniques for the treatment of femur fracture in patients with osteopetrosis. Generally, patients with osteopetrosis tend to be highly resistant to recovery and often display many complications after operation. However, through the improvement of preoperative planning, surgical techniques and postoperative rehabilitation nursing, complications of surgical treatment for osteopetrosis-associated fractures can be avoided. We cannot deny that the bone condition of patients with osteopetrosis is special, but its treatment philosophy is consistent with that of other fractures. Whether treatment is conservative or surgical, we should make full use of the advantages of each method. On the whole, owing to the large differences of bone circumstances in patients with osteopetrosis, clinicians should pay extreme attention to preoperative assessment and preparation so as to select the most appropriate surgical method for patients. Secondly, osteopetrotic fractures are obstinate, and surgery can be prolonged and tough, emphasizing the importance of surgical techniques (Table 3). Last but not least, considering the healing of osteopetrotic subtrochanteric fractures takes a long time, the mechanical stabilization of internal fixation and avoiding trauma after operation are critical, indicating that external fixation after operation is effective and necessary.

## Data Availability

The datasets used and analyzed during the current study are available from the corresponding author on reasonable request.

## Abbreviations

IRO: Intermediate autosomal recessive osteopetrosis
DHS: Dynamic hip screw
ARO: Autosomal recessive osteopetrosis
ADO: Autosomal dominant osteopetrosis
XLO: X-linked osteopetrosis
DCS: Dynamic condylar screws
IMN: Intramedullary nail
PFNA: Proximal femoral anti-rotation intramedullary nail
LCP: Locking compression plate
LISS: Less invasive stabilization system
THA: Total hip arthroplasty

## References

[1] RoviraMartí P, UllotFont R (2016). Orthopaedic disorders of pycnodysostosis: a report of five clinical cases. Int Orthop.

[2] Palagano E, Menale C, Sobacchi C, Villa A (2018). Genetics of osteopetrosis. Curr Osteoporos Rep.

[3] Elster AD, Theros EG, Key LL, Chen MY (1992). Cranial imaging in autosomal recessive osteopetrosis. Part I. Facial bones and calvarium. Radiology.

[4] Fattore DA, Cappariello A, Teti A (2008). Genetics, pathogenesis and complications of osteopetrosis. Bone.

[5] George A, Zand DJ, Hufnagel RB, Sharma R, Sergeev YV, Legare JM, Rice GM, ScottSchwoerer JA, Rius M, Tetri L, Gamm DM, Bharti K, Brooks BP (2016). Biallelic mutations in MITF cause coloboma, osteopetrosis, microphthalmia, macrocephaly, albinism, and deafness. Am J Hum Genet.

[6] Kleinberg S (1954). Osteopetrosis. Am J Surg.

[7] Kahler SG, Burns JA, Aylsworth AS (1984). A mild autosomal recessive form of osteopetrosis. Am J Med Genet.

[8] Birmingham P, McHale KA (2008). Case reports: treatment of subtrochanteric and ipsilateral femoral neck fractures in an adult with osteopetrosis. Clin Orthop Relat Res.

[9] Chhabra A, Westerlund LE, Kline AJ, McLaughlin R (2005). Management of proximal femoral shaft fractures in osteopetrosis: a case series using internal fixation. Orthopedics.

[10] Strickland JP, Berry DJ (2005). Total joint arthroplasty in patients with osteopetrosis: a report of 5 cases and review of the literature. J Arthroplasty.

[11] Bhargava A, Vagela M, Lennox CM (2009). "Challenges in the management of fractures in osteopetrosis"! Review of literature and technical tips learned from long-term management of seven patients. Injury.

[12] Nampei A, Hashimoto J (2009). Bone fracture and the healing mechanisms Metabolic bone disease and skeletal healing. Clin Calcium.

[13] Amit S, Shehkar A, Vivek M, Shekhar S, Biren N (2010). Fixation of subtrochanteric fractures in two patients with osteopetrosis using a distal femoral locking compression plate of the contralateral side. Eur J Trauma Emerg Surg.

[14] Huang J, Pan J, Xu M, Xu S (2017). Successful open reduction and internal fixation for displaced femoral fracture in a patient with osteopetrosis: case report and lessons learned. Medicine.

[15] Seyfettinoglu F, Tuhanioğlu Ü, Ogur HU, Cicek H (2016). Proximal femoral fracture surgery in a patient with osteopetrosis tarda: complications and treatment strategy. Int Med Case Rep J.

[16] Beighton P, Hamersma H, Cremin BJ (1979). Osteopetrosis in South Africa. The benign, lethal and intermediate forms. S Afr Med J.

[17] Horton WA, Schimke RN, Iyama T (1980). Osteopetrosis: further heterogeneity. J Pediatr.

[18] Ramiah RD, Baker RP, Bannister GC (2006). Conversion of failed proximal femoral internal fixation to total hip arthroplasty in osteopetrotic bone. J Arthroplasty.

[19] Matsuno T, Katayama N (1997). Osteopetrosis and total hip arthroplasty. Report of two cases. Int Orthop.

[20] Yang BJ, Chen CF, Lien IN (1980). Rehabilitation of left femur subtrochanteric fracture in osteopetrosis—a case report. Taiwan yi xue hui za zhi.

[21] Ashby ME. Total hip arthroplasty in osteopetrosis. A report of two cases. Clin Orthopaed Relat Res. 1992:214–21. 1537156

[22] de Palma L, Tulli A, Maccauro G, Sabetta SP, del Torto M. Fracture callus in osteopetrosis. Clin Orthopaed Relat Res. 1994:85–9. 7955707

[23] Armstrong DG, Newfield JT, Gillespie R (1999). Orthopedic management of osteopetrosis: results of a survey and review of the literature. J Pediatr Orthop.

[24] Rolauffs B, Bernhardt TM, von Eiff C, Hart ML, Bettin D (2002). Osteopetrosis, femoral fracture, and chronic osteomyelitis caused by *Staphylococcus aureus* small colony variants (SCV) treated by girdlestone resection—6-year follow-up. Arch Orthop Trauma Surg.

[25] Su YJ, Chiang WK, Chang KS (2003). Chalk bones and pathological fractures: case report and review of the literature. J Emerg Med.

[26] Zhang ZF, Wang D, Wu LD, Dai XS (2017). Case report: A 10 years follow-up of periprosthetic femoral fracture after total hip arthroplasty in osteopetrosis. Chin J Traumatol.

[27] Cadosch D, Gautschi OP, Brockamp T, Zellweger R (2009). Osteopetrosis—a challenge for the orthopaedic surgeon. S Afr J Surg.

[28] Kumar D, Jain VK, Lal H, Arya RK, Sinha S (2012). Metachronous bilateral subtrochanteric fracture of femur in an osteopetrotic bone: a case report with technical note. J Clin Orthop Trauma.

[29] Golden RD, Rodriguez EK (2010). Management of subtrochanteric femur fractures with internal fixation and recombinant human bone morphogenetic protein-7 in a patient with osteopetrosis: a case report. J Med Case Rep.

[30] Sonohata M, Okubo T, Ono H, Mawatari M, Hotokebuchi T (2011). Bipolar hip arthroplasty for subtrochanteric femoral nonunion in an adult with autosomal dominant osteopetrosis type II. J Orthop Sci.

[31] Kunnasegaran R, Chan YH (2017). Use of an industrial tungsten carbide drill in the treatment of a complex fracture in a patient with severe osteopetrosis: a case report. Malays Orthop J.

[32] Sen RK, Gopinathan NR, Kumar R, Saini UC (2013). Simple reproducible technique in treatment for osteopetrotic fractures. Musculoskelet Surg.

[33] Huang T, Liang Q, Qian H, Li X, Zou C (2013). Surgical treatment of an osteopetrotic patient with postoperative fractures: lessons from siblings with osteopetrosis. Tohoku J Exp Med.

[34] Kumbaraci M, Karapinar L, Incesu M, Kaya A (2013). Treatment of bilateral simultaneous subtrochanteric femur fractures with proximal femoral nail antirotation (PFNA) in a patient with osteopetrosis: case report and review of the literature. J Orthop Sci.

[35] Matar HE, James LA. A challenging paediatric pathological femur fracture in pyknodysostosis (osteopetrosis acro-osteolytica): lessons learnt. BMJ Case Rep. 2014;2014. 10.1136/bcr-2014-207730 PMC4244497 25414231

[36] Behera P, Khurana A, Saibaba B, Aggarwal S (2016). Dealing with sub-trochanteric fracture in a child with osteopetrosis: a case report. Acta Orthop Belg.

